# Atorvastatin for reduction of 28-day mortality in severe and critical COVID-19 patients: a randomized controlled trial

**DOI:** 10.1186/s12931-024-02732-2

**Published:** 2024-02-22

**Authors:** Naglaa Hamdi Eltahan, Neamat Hamdy Elsawy, Kholoud M. Abdelaaty, Amal Salah Elhamaky, Ahmed H. Hassan, Moataz Maher Emara

**Affiliations:** 1 Sherbin Central Hospital, Ministry of Health and Population, Sherbin, Egypt; 2 Fowa Health District, Preventive Sector, Epidemiology and Surveillance Department, Ministry of Health and Population, Fowa, Egypt; 3 Department of Clinical Research, Fowa Central Hospital, Ministry of Health and Population, Fowa, Egypt; 4 Mansoura University, Specialized Medical Hospital, Mansoura, Egypt; 5 Mansoura Specialized Hospital, Mansoura, Ministry of Health and Population, Mansoura, Egypt; 6 Mansoura University, Mansoura University Hospital, Mansoura, Egypt; 7 Department of Anesthesiology and Intensive Care and Pain Medicine, Mansoura University Faculty of Medicine, 60 Elgomhoria St, Mansoura, 35516 Egypt

**Keywords:** COVID-19, SARS-CoV-2, Coronavirus, Statins, hmg coa, Atorvastatin, Mortality

## Abstract

**Background:**

COVID-19 is an abnormal host response to the SARS-CoV-2 infection, which is associated with endothelial dysfunction and multi-organ failure. Atorvastatin has been proposed to reduce COVID-19 severity and mortality in chronic and de-novo users.

**Methods:**

This randomized double-blind trial included 220 COVID-19 patients admitted to Mansoura University's isolation hospital in Egypt. One hundred and ten cases were given 40 mg of atorvastatin once daily for 28 days (group A), while 110 received a placebo (group B). All patients received treatment as per hospital protocol. The primary outcome is all-cause mortality at 28 days. We also tracked 6-month mortality, time to clinical improvement, the risk of invasive mechanical ventilation, acute kidney injury, potential adverse events, and hospital and intensive care length of stay.

**Results:**

The 28-day all-cause mortality was 52/104 (50%) in group A vs. 54/103 (52.4%) in group B, odds ratio (OR) = 0.907 (0.526, 1.565), P = 0.727; adjusted OR = 0.773 (0.407, 1.47), *P* = 0.433. Six-month mortality occurred in 53/102 (52%) and 59/79 (60.8%) in group A vs. B, respectively, *P* = 0.208. Among hospital survivors in group A vs. group B, the median time to clinical improvement was 10 days (7–14) vs. 10 (7–15), *P* = 0.715; the duration of hospital stay was 10 days (7–14) vs. 10 (8–17), *P* = 0.378. Discontinuation was higher in group B (four vs. one), but statistically insignificant, *P* = 0.369.

**Conclusions:**

In adults with severe or critical COVID-19, atorvastatin did not reduce the risk of 28-day or 6-month mortality and did not shorten the length of hospital stay or time to clinical improvement.

*Trial registration* Clinical Trial Registry (NCT04952350) on July 1st, 2021. https://clinicaltrials.gov/ct2/show/NCT04952350

**Supplementary Information:**

The online version contains supplementary material available at 10.1186/s12931-024-02732-2.

## Introduction

Despite widespread vaccination against SARS-CoV-2, the virus has not been eradicated, and COVID-19 has transitioned from pandemic to endemic status. COVID-19 denotes a cytokine storm, implying an aggressive immune response [1]. Increased cytokines like interleukin-6 (IL-6) activate the nuclear factor kappa B (NF-B) pathway, which causes sepsis, capillary damage, acute pulmonary injury, severe acute respiratory distress (ARDS), multi-organ damage, and death [2, 3].

Statins are lipid-lowering medications that also have pleiotropic effects. Their antiviral, anti-inflammatory, antithrombotic, and antioxidant properties may benefit COVID-19 patients: (1) reduce viral load by activating autophagy and regulating SARS-CoV-2 virus degradation or replication [4]; (2) modulate the cytokine storm by blocking the NF-B pathway and NLRP3 inflammasomes [4]; (3) improve endothelial function, which contributes to COVID-19 pathogenesis [5]; (4) enhance vein thrombus resolution and reduce the risk of recurrent pulmonary embolism (PE) [5, 6].

Retrospective studies suggest that chronic statins reduce the risk of mortality, invasive mechanical ventilation, and hospitalization duration [7–9]. Recent randomized trials, however, have yielded contradictory results. The INSPIRATION-S trial found no benefit for 20 mg atorvastatin for 30 days in COVID-19 patients admitted to intensive care [10], and the RESIST trial found the same with 40 mg atorvastatin for 10 days or hospital discharge in mild-to-moderate cases [11]. Another study, on the other hand, claimed a shorter hospital stay and lower C-reactive protein levels with statins [12]. Furthermore, a recent review that summarized the current evidence clarified this debate [13].

Consequently, we designed a randomized clinical trial to investigate the role of atorvastatin in hospitalized (severe and critical cases) COVID-19 patients on the 28-day mortality, clinical improvement, and 6-month mortality. We hypothesized that atorvastatin 40 mg once daily for 28 days would reduce the 28-day all-cause mortality in adult hospitalized COVID-19 patients.

## Methods

We conducted this randomized (1:1) parallel controlled superiority trial at Mansoura University COVID-19 Isolation Hospital, Mansoura City, Egypt. According to the declaration of Helsinki, we obtained institutional review board approval (R.21.04.1300) on April 2nd, 2021and registered the study at clinical trial.gov (NCT04952350) on July 1st, 2021. We obtained written informed consent from patients or the patient’s legal representative if the patient was unable to give consent. We wrote this manuscript according to the CONsolidated Standards of Reporting Trials (CONSORT) guidelines [14]. For detailed methodology and related documents, the reader can find the published protocol [15].

Two hundred and twenty adult patients (≥ 18 years old) with severe or critical COVID-19 who were admitted to the Mansoura University isolation hospital—from 18th August 2021 to 6th October 2021—were included in the study. We included patients who had COVID-19 diagnosed by PCR, clinically or radiologically. PCR was then used to confirm all cases.

According to the WHO definition [16], cases are classified as severe or critical, with severe cases having clinical signs of severe pneumonia and SpO2 < 90% on room air or RR > 30 breaths/min without any critical criteria. While critical cases have ARDS, sepsis, septic shock, pulmonary embolism, acute coronary syndrome, or acute stroke.

Exclusion criteria included chronic statin use, serum creatine kinase (CK) levels greater than 5 times the upper limit of normal (ULN), serum transaminases greater than 5 times the ULN, acute hepatic failure, chronic liver disease with Child–Pugh Classification C, a history of rhabdomyolysis or myopathies, or severe renal impairment not receiving renal replacement therapy (estimated Cr cl 30 ml/min). Additionally, we excluded pregnant and lactating women, patients who are expected to die within 48 h, or patients on cyclosporine, ritonavir, or chronic colchicine.

### Randomization and allocation concealment

A randomization Table (1:1) was created by a pharmacist who was not involved in the study using computer-generated random permuted blocks (4, or 6 in each block). The same pharmacist assigned patients to either group without providing the study group with the randomization table. After evaluating the inclusion criteria, randomization was carried out within 24 h of hospital admission.

For 28 days, patients were randomly assigned to receive either atorvastatin (*Atorstat, Delta Pharm Co., Egypt*) 40 mg/day orally (group A) or placebo (group B). During the hospitalization, all cases received corticosteroids and prophylactic anticoagulation. All patients received medical care according to the local hospital protocol and at the discretion of the treating physician. The placebo is similar to the original drug in terms of the drug package, tablet color, consistency, and size. Unconscious or ventilated patients received the drug through a nasogastric tube.

### Blinding

Patients, caregivers, data collectors, and data analysts were all blinded to the study group. Delta Pharma (Egypt) supplied the atorvastatin and a similar placebo. The company did not participate in any aspect of the study, including participant recruitment, data collection, data analysis, or results reporting. The company prepared a similar drug and placebo package (labeled as A and B). Even the pharmacist, who is involved in treatment allocation, had no idea about what treatment group is—only A or B. In this manuscript, we used “A” to describe the Atorvastatin group and “B” to describe the placebo group. This is not necessarily the drug labels used in the study.

### Criteria for discontinuation

We daily monitored patients for adverse events; if the patient developed myalgia or unexplained weakness, we checked CK levels and stopped if CK was > 10 times ULN, drug-induced hepatitis, or serum transaminases were > 8 times ULN. Patients who received azole antifungals had a lower monitoring threshold for liver injury and rhabdomyolysis.

According to local hospital protocol, all patients received the standard of care. Antiviral therapy was permitted.

### Data safety and monitoring board

The assignment groups were hidden from the Data Safety and Monitoring Board (DSMB). The true labels and the randomization table were kept in a closed envelope for emergency unmasking by one of the directors of the Mansoura COVID-19 research council who was not participating in the study. The envelope was only supposed to be opened if the DSMB decided to unmask it for safety reasons; however, unmasking was not required during this study.

### Measured outcomes

The primary outcome was mortality from any cause within 28 days of randomization. Patients or their relatives were contacted by the trial staff to collect this data.

The main Secondary outcomes were 6-month mortality, in-hospital mortality, need for invasive mechanical ventilation (Yes/No binary outcome), oxygen support duration (days), time to clinical improvement (defined as 2 points reduction in the WHO disease ordinal progression scale [17] or discharge, whatever came first), and ICU and hospitalization length of stay in survived patients (days). Apart from the six-month mortality, these data were collected for the duration of the hospitalization.

Additional outcomes included: (1) serious adverse effect resulting in drug discontinuation; (2) CRP on days 3, 7, 14, and 28; (3) SOFA score on days 3, 7, 14, and 28; (4) COVID19 WHO disease progression scale on days 3, 7, 14, and 28; and (5) incidence of acute kidney injury (AKI), defined as an increase in Scr of 0.3 mg/dL in 48 h or 50% increase in Scr in 7 days or Oliguria for 6 h [18]. We reported data about CRP, d-dimers, SOFA scores, WHO disease progression score, and AKI only during the hospitalization period. Venous thromboembolism was collected according to the suspicion of the treating physician and the subsequent appropriate investigations.

We did not change the study outcomes after the commencement of the study; however, we did not collect the long-term outcomes except the six-month mortality.

### Sample size

A sample size of 97 patients in each group would achieve at least 80% power to detect a risk difference of 0.2 (20%) in the 28-day all-cause mortality (primary outcome) between the null hypothesis (both proportions are 0.6) and the alternative hypothesis (the proportion in the non-statin group would be 0.4). In the published protocol, we explained in detail why we chose 0.2 as a risk difference in mortality for sample size calculation [15].

We assumed a significance level (α) of 0.05 and used the Chi-square test of independent proportions in MedCalc software. To account for the estimated loss-to-follow-up, we increased the sample size in each group to 110 patients.

### Statistical analysis plan

The modified intention-to-treat strategy with available case analysis is used in the primary analysis. Categorical variables are presented as percentages and proportions. For parametric data, continuous variables are presented as a mean (standard deviation) or as a median (25th–75th percentile) for non-parametric data. In the event of missing data, we reported the denominator or the number (percentage) in the tables or text, as appropriate. The SAMPL guidelines were followed in reporting the statistical results [19].

Univariate analysis of demographic features correlated with study groups was performed using the Chi-square test and the t-test, reporting the 95% confidence interval and the P-value. We compared the 28-day all-cause mortality rate (primary outcome) in each group using the chi-square test, reporting the rate ratio and 95% confidence interval.

SPSS version 28 was used for statistical analysis. The statistical significance level is p-value 0.05.

### Interim analyses

We conducted interim analyses of in-hospital mortality after recruiting 25, 50, and 75% of the planned sample size. The DSMB and IRB members were blinded to the study allocation and were kept up to date on the results of the interim analyses. The results of the interim analyses were published with the protocol [15].

### Subgroup analyses and regression analyses

As pre-planned, we performed a subgroup analysis of the 28-day mortality and 6-month mortality by severity, as defined 48 h after recruitment. Subgroup analysis by invasive mechanical ventilation (IMV) was not performed due to the very low event rate of IMV after 48 h of recruitment (eight cases in group A and ten cases in group B).

We used multiple logistic regression to test the association of atorvastatin use and 28-day and 6-month all-cause mortality while controlling for gender, age, comorbidities (HTN, AF, IHD, DM, or COPD), and COVID-19 severity (critical or severe). Severity was defined According to the WHO definition [16]. The assumptions assessed using the binary logistic regression models were the linearity of the continuous predictors using the Box–Tidwell approach, independence, absence of multicollinearity—the variance inflation factor (VIF < 5), and residual outliers. We presented the adjusted and unadjusted odds ratios (OR) in Table 3, reporting the 95% CI and R^2^ (the coefficient of multiple determination of the model).

We did not report the reduction in the invasive mechanical ventilation duration as survival in this group was low in both groups (11 cases in group A and 13 cases in group B).

As an exploratory analysis, we stratified the effect of atorvastatin on 28-mortality the time between symptom onset and hospitalization (within 7 days vs > 7 days).

## Results

**Fig. 1:**
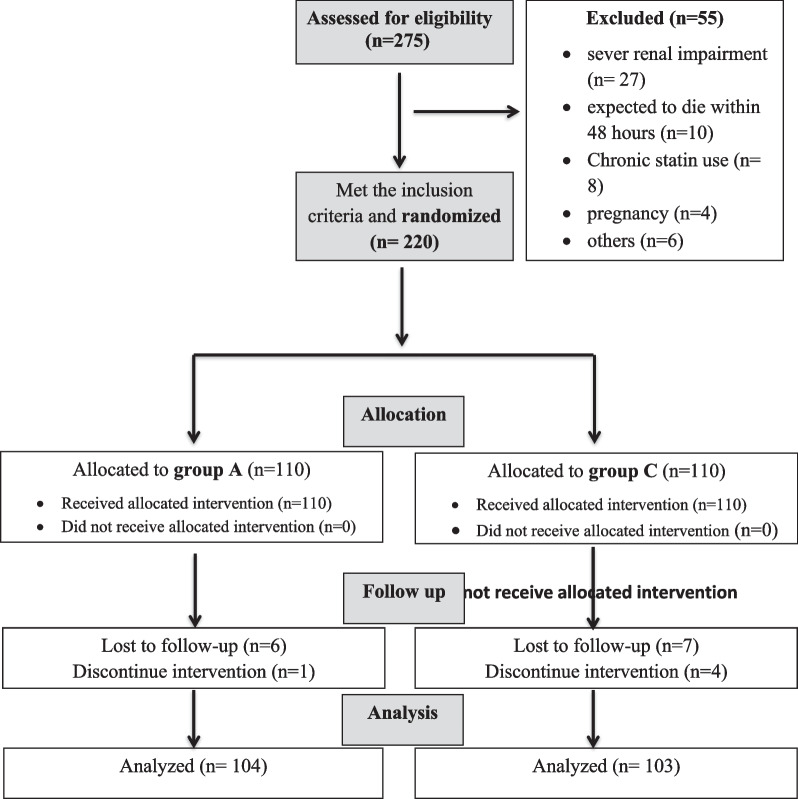
Study CONSORT flowchart

As shown in the participant flowchart (Fig. 1), 275 COVID-19 hospitalized patients were screened for eligibility, and 220 patients were randomly assigned to two equal groups. Baseline characteristics and laboratory results are shown in Table 1. Additionally, we depicted the patients’ presentation on admission in Additional file 1: Table S1. SARS-CoV-2 positivity was confirmed by PCR in all cases.

**Table 1:** Baseline characteristics

	Group A (n = 110)	Group B (n = 110)
Age	61.4 (13)	60.8 (12.7)
Male no. (%)	51 (46.4%)	45 (40.9%)
Weight (kg)	76.7 (12)	74 (10.7)
Not SARS-CoV-2 vaccinated	105 (98.1%)	107 (98.2%)
Critical cases on admission	21 (19.1%)	20 (18.2%)
WHO score on admission	5 (5–6)	5 (5–6)
Smokers	16 (14.5%)	13 (11.8%)
CLD	8 (7.4%)	7 (6.5%)
Hypertension	50 (45.9%)	46 (41.8%)
Cardiac disease	14 (13%)	13 (11.8%)
Diabetes mellitus	46 (42.6%)	39 (35.8%)
ESLD	9 (8.5%)	10 (9.1%)
ESRD	2 (1.8%)	1 (0.9%)
Neurological disease	6 (5.6%)	5 (4.6%)
Basal GCS	15 (15–15)	15 (15–15)
Basal SOFA	2 (1–3)	2 (1–3)
AST (U/L)	40 (25.25–59.5)	36 (25.5– 51.5)
ALT (U/L)	33 (18–61.75)	31 (17–52)
Total Bilirubin (mg/dL)	0.54 (0.33–78)	0.46 (0.36–0.7)
INR	1.1 (1.01–1.22)	1.13 (1.03 –1.22)
Albumin (g/dL)	3.2 (2.9–3.5)	3.1 (2.9–3.5)
S.Cr (mg/dL)	0.96 (0.8–1.25)	0.9 (0.7–1.27)
CK (IU/L)	75 (40–187.5)	61 (36–163.5)
Hemoglobin (g/dL)	12.8 (11.54–13.9)	12.48 (11.17–13.83)
TLC (10^3^/µL)	7.9 (5.79–9.97)	8.93 (5.94–12.68)
Lymph (10^3^/µL)	0.83 (0.52–1.14)	0.92 (0.59–1.51)
Platelets (10^3^/µL)	197.5 (135.5–265.55)	211.5 (149.75–284)
LDH (IU/L)	311.5 (254–407.75)	369 (252.25–509.5)
D-dimer (g/L)	0.2 (0–0.3)	0.2 (0–0.4)
CRP (mg/L)	96 (44–143)	96 (27–129)
Ferritin (µg/L)	514.5 (315.5–543.5)	529 (370–546)

Data presented as mean (SD), median (P_25th_–P_75th_), or number (%)

Group A: Atorvastatin group; Group B: Placebo group; Cardiac disease: history of Atrial fibrillation of Ischemic heart not on Statins; ESLD: End stage Liver Disease; ESRD: End Stage Renal Disease; CLD: Chronic Lung Disease—COPD or Bronchial Asthma; GCS: Glasgow Coma Score; SOFA: Sequential Organ Failure Assessment; Neurological disease implies dementia, stroke, multiple sclerosis, Parkinson, or psychosis; AST: Aspartate Transaminase; ALT: Alanine Transaminase; INR: International Normalized Ratio; S.Cr: Serum creatinine; CK: Creatine kinase; TLC: Total Leucocytic count; LDH: Lactate Dehydrogenase; CRP: C-Reactive protein

### Primary outcome and main outcomes

The 28-day all-cause mortality did not differ statistically between groups, 52/104 (50%) in group A vs 54/103 (52.4%) in group B, RR = 0.954, 95% CI 0.731, 1.244, *P* = 0.727. Likewise, the in-hospital and 6-month mortality did not show any difference (Table 2).

**Table 2:** Main outcomes

	Group A (n = 110)	Group B (n = 110)	P-value	Risk ratio (95% CI)
Need for IMV	38/107 (35.5%)	43/106 (40.6%)	0.448	0.874 (0.685–1.18)
Duration of O2 support^#^	7 (5–12)	8 (6–15.75)	0.098	
AKI	9 (8.3%)	13 (11.8%)	0.381	0.7 (0.57–1.21)
In hospital mortality	45 (40.9%)	49 (44.5%)	0.586	0.919 (0.713–1.21)
28-day mortality	52/104 (50%)	54/103 (52.4%)	0.727	0.95 (0.731–1.244)
Six-month mortality	53/102 (52%)	59/97 (60.8%)	0.208	0.854 (0.668–1.092)

Data presented as mean (SD), median (P_25th_–P_75th_), or number (%)

Group A: Atorvastatin group; Group B: Placebo group; IMV: Invasive Mechanical Ventilation; ICU: Intensive Care Unit; AKI: Acute Kidney Injury; 95% CI: 95% Confidence Interval

^*^P ≤ 0.05 indicated statistical significance

^#^In survived patients till discharge, Data is analyzed based on available case analysis

Among hospital-survived in group A vs B, the median of the time to clinical improvement was 10 (7–14) vs 10 (7–15), *P* = 0.715; the duration of hospital stay was 10 (7–14) vs 10 (8–17), *P* = 0.378; the duration of ICU stay was 7 (5–16) vs 10 (6–16), *P* = 0.288. As shown in Table 2, the need for invasive mechanical ventilation did not differ significantly between groups.

**Fig. 2:**
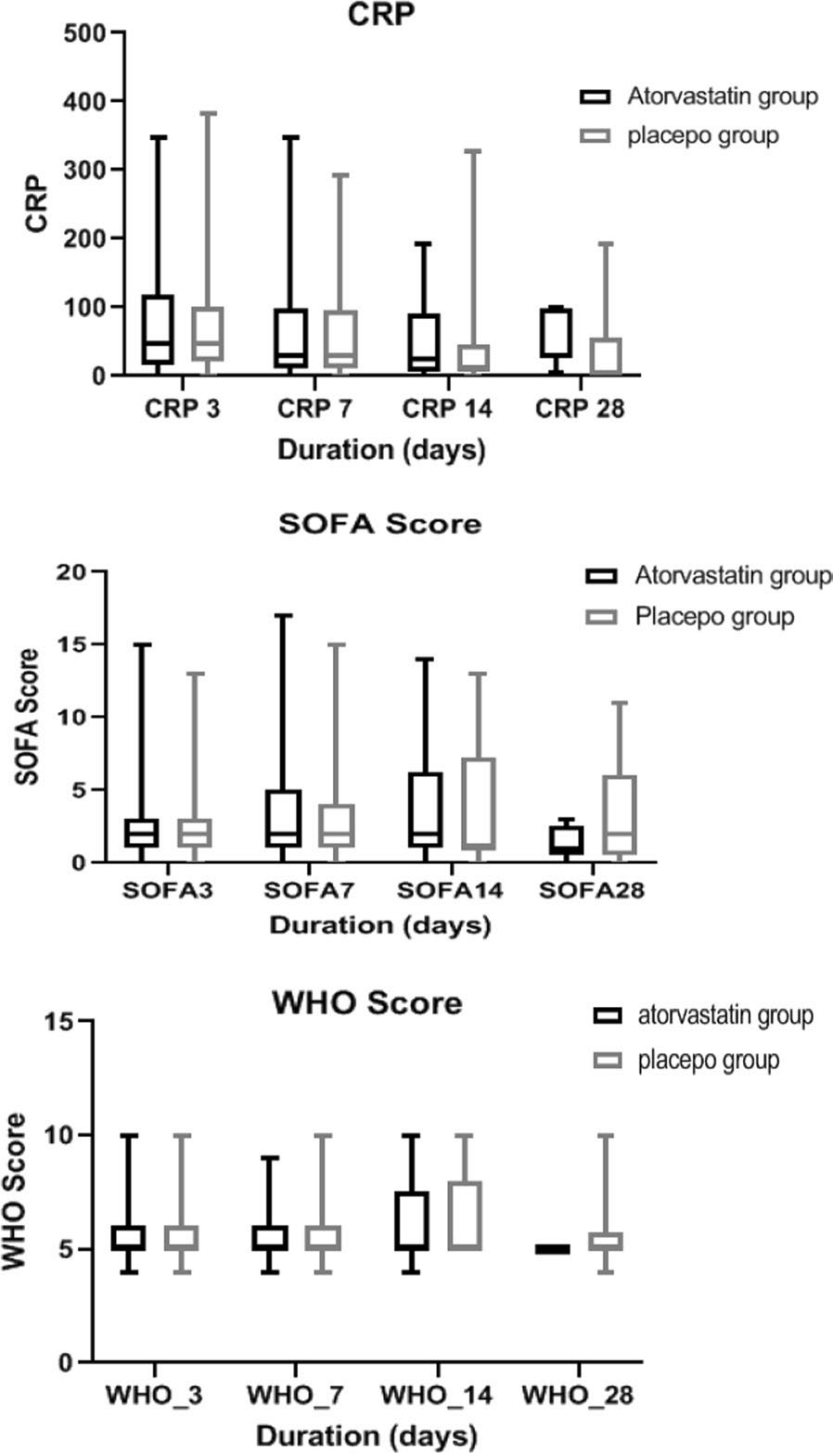
CRP, SOFA, and WHO scores

In this cohort, there were no cases of DVT or pulmonary embolism. Major outcomes are presented in Table 2. Figure 2 and Additional file 1: Table S2 show the non-statistical differences of in-hospital CRP, SOFA score, and WHO score in the 3rd, 7th, 14th, and 28th days after admission. Unfortunately, we could not retrieve the long-term outcomes other than the six-month mortality.

### The onset of symptoms to admission duration

The median time from onset of symptoms to admission in the entire sample was 7 days (4–9); 6 days (4–8) vs 7 days (4–10) in groups A and B, respectively; *P* = 0.411.

### The discontinuation rate and adverse events

The discontinuation rate was 1 (0.9%) in group A vs 4 (3.6%) in group B, *P* = 0.369. The Atorvastatin group had one participant who had an elevation of liver transaminase more than 10 times the upper limit of the normal range, while the control group had four discontinuation events: two cases had an elevation of liver transaminases, and two cases had a rise in creatine kinase rise and liver transaminases. We did not find harm or unintended effects during the study.

Treatment compliance was 100% in all patients during the in-hospital period, with adherence until 28 days or mortality confirmed in 79 (72%) and 84 (76%), respectively, in groups A and B; *P* = 0.442.

### In-hospital management

Almost all patients received steroids and anticoagulation during hospitalization. The summary of the management of cases is provided in the Additional file 1: Table S3 and showed no statistical differences between both groups.

### Subgroup analyses

As shown in Additional file 1: Tables S4 and S5, atorvastatin did not reduce 28-day mortality in subgroups, severe and critical cases—taking into account the small number of critical cases, 20 and 17 cases in groups A and B, respectively. We did not perform the subgroup analysis of the mechanically ventilated versus non-mechanically ventilated patients due to the very low event rate of mechanical ventilation in the first 48 h after recruitment, as predefined in the protocol.

In the exploratory analysis, patients admitted to the hospital within seven days of the onset of symptoms had an RR of 28-day mortality of 0.959, 95% CI 0.609, 1.512; when admitted after 7 days, the RR of 28-day mortality was 0.736, 95% CI 0.402, 1.348; Mantel–Haenszel *P* value = 0.558.

### Regression analyses

Binary multivariable logistic regression showed that patients on atorvastatin showed 0.773 (95% CI 0.407, 1.47) non-significant lower odds of suffering 28-day mortality after controlling for confounders: age, sex, number of comorbidities, and disease severity, compared to those who did not take atorvastatin. Model 1 showed statistical significance [X2 (df = 5) = 63.209, P < 0.001], explaining 35.1% (Nagelkerke R2) of the variance in 28-day mortality and correctly diagnosing 73.9% of the cases (Table 3). The results of the multivariable logistic regression on the six-month mortality are shown in Table 3.

**Table 3:** Bivariate and multivariable analyses of Atorvastatin use and 28-day mortality or 6-month mortality

Independent factors	Bivariate at 28-day mortality		Multivariable model at 28-day mortality		Bivariate at 6-month mortality		Multivariable model at 6-month mortality	
	Unadjusted OR (95% CI)	*P* value	Adjusted OR (95% CI)	*P* value	Unadjusted OR (95% CI)	*P* value	Adjusted OR (95% CI)	*P* value
Atorvastatin	0.907 (0.526, 1.565)	0.727	0.773 (0.407, 1.47)	0.433	0.697 (0.397, 1.223)	0.208	0.573 (0.293, 1.117)	0.102
Age	1.065 (1.037, 1.094)	< 0.001*	1.078 (1.046, 1.11)	< 0.001*	1.061 (1.033, 1.09)	< 0.001*	1.077 (1.044, 1.11)	< 0.001*
Male	1.862 (1.043, 3.325)	0.036*	1.881 (0.978, 3.618)	0.058	1.569 (0.864, 2.849)	0.139	2.185 (1.104, 4.324)	0.025*
No. of comorbidities	1.076 (0.801, 1.444)	0.627	0.856 (.605, 1.212)	0.382	1.078 (0.794, 1.464)	0.629	0.802 (0.557, 1.154)	0.234
Critical cases	0.082 (0.024, 0.278)	< 0.001*	14.343 (4.263, 48.252)	< .001*	17.5 (4.062, 75.388)	< 0.001*	17.903 (4.516, 70.979)	< 0.001*

OR: odds ratio; CI: confidence interval. No. of comorbidities includes (Diabetes mellitus, hypertension, ischemic heart disease, AF, Chronic obstructive pulmonary disease)

^*^P ≤ 0.05 indicated Statistical significance. The constant of the 28-day mortality model is − 4.855, while the constant of the 6-month mortality model is − 4.456

## Discussion

In this double-blind randomized controlled trial of adult patients with COVID-19 admitted to the ICU, atorvastatin 40 mg once daily for 28 days—compared with placebo—did not reduce the 28-day mortality. Likewise, atorvastatin did not reduce the risk of major secondary outcomes like in-hospital mortality, 6-month mortality, AKI, or IMV.

Like our results, a randomized clinical trial showed that a lower dose of atorvastatin (20 mg) once daily in ICU patients with COVID‐19 for 30 days did not reduce the composite outcome of venous or arterial thrombosis, treatment with extracorporeal membrane oxygenation, or all‐cause mortality [10]. The authors supposed that the effect of atorvastatin on thrombosis or mortality may be shown beyond the 30-day follow‐up; therefore, they plan to extend the follow-up for 90 days [10]. Another randomized trial of non-critically ill hospitalized patients with COVID-19 reported that the combination of colchicine and rosuvastatin in addition to standard of care didn’t appear to reduce the risk of progression of COVID-19 disease or thromboembolic events [20]. More recently, the international REMAP-CAP study did not find a superiority of simvastatin over the control regarding the cardiovascular and respiratory organ support-free days in COVID-19 patients [21].

In COVID-19, early statin administration may be critical for the beneficial statin effects. As a result, studies that looked at outcomes in hospitalized patients who were already taking statins found that they had a higher chance of survival [7, 22]. In addition, a prospective trial found a marginal survival benefit in patients who started atorvastatin within seven days of symptom onset [10]. On the other hand, we did not find that the time of symptom onset at the time of hospitalization interacts with the effect of statins on mortality—in the exploratory strata analysis.

In contrast to our results, a randomized controlled trial claimed that adding atorvastatin 40 mg to the standard regimen of lopinavir/ritonavir decreased hospital stays (8 vs. 10 days, *P* = 0.01) and CRP levels (*P* = 0.01) [12]. However, this trial included only 40 participants—vs 220 in our study—and administered atorvastatin for 5 days vs 28 days in our trial [12]. Another observational retrospective study reported a slower progression to death associated with atorvastatin in patients with COVID-19 admitted to ICU [9]. On the other side, one randomized controlled trial suggested that 20 mg atorvastatin per day increased the length of hospital stay (4 days vs. 6, *P* = 0.001) and the risk of ICU admission (18.4% vs. 1.3%) [23].

A high dose of atorvastatin (40 mg) appears to be safe in severe COVID-19 cases. The placebo group had four patients drop out, whereas the Atorvastatin group had one patient experience a significant increase in liver enzymes. Similarly, the INSPIRATION-S reported that atorvastatin was safe in COVID-19 intensive care patients [10]. This may be not true with simvastatin, as the REMAP-CAP study reported increased CK and liver enzyme levels [21]. Due to limited resources, we were unable to perform serial laboratory monitoring for CK level. There were no detected adverse events in the form of myalgia or unexplained weakness.

In our sample, the 28-day mortality rate was relatively high. Other studies, however, have shown similar rates early and throughout the pandemic [24, 25]. Additionally, we only included severe and critical patients. Throughout the pandemic, our center has been overloaded with severe and critical cases.

We did not report any cases of DVT or PE in either group in our study. The low rates of thromboembolism could be attributed to the routine use of prophylactic anticoagulation in severe COVID-19 cases. We assume, however, that randomization balanced these factors, and that any missed cases were clinically insignificant. Like the RESIST trial, we did not find differences between groups regarding the in-hospital CRP levels, SOFA, or WHO scores. However, the RESIST trial only included mild and moderate COVID patients [11]. The study has some limitations: first, we missed some follow-ups, but this was successfully compensated for during sample size estimation; and second, patient management was not standardized, and we only intervened with our study drug. We believe that pragmatic management does not affect the conclusions' validity: randomization would have balanced the management changes, and standardization of care in such cases during the pandemic was difficult. Finally, our study targeted severe and critical cases of COVID-19; therefore, our findings are not generalizable to mild or moderate cases.

The strengths of the current study include the randomized controlled and double-blind design that reduced the risk of allocation and performance bias; the inclusion of DSMB; the long-term follow-up (6 months); and the published protocol [15].

Furthermore, future studies would look into the effect of statins on subgroups (severe or critical cases).

In conclusion, in adults with severe or critical COVID-19, atorvastatin did not reduce the risk of 28-day mortality or 6-month mortality. Additionally, atorvastatin did not improve the hospital or ICU length of stay, AKI, or oxygen therapy duration.

### Supplementary Information

**Additional file 1: Table S1.** Presentation on admission. Data presented as mean (SD), median (P_25th_–P_75th_), or number (%).**Table S2.** CRP, WHO, SOFA scores in survived in-hospital patients. Data presented as mean (SD), median (P_25th_–P_75th_), or number (%). **Table S3.** Drugs and Interventions Offered to patients in both groups. **Table S4.** 28-mortality by severity. **Table S5.** Six-month mortality by severity.

## Data Availability

Individual data will be available with the corresponding author upon reasonable request after approval of the local institutional review board.

## Abbreviations

AF: Atrial fibrillation
AKI: Acute kidney injury
ARDS: Acute respiratory distress syndrome
CARDS: COVID-19 ARDS
CK: Creatine kinase
COPD: Chronic obstructive pulmonary disease
COVID-19: Coronavirus disease of 2019
CRF: Case report form
DM: Diabetes mellitus
DSMB: Data and Safety Monitoring Board
HMG-CoA: β-Hydroxy β-methylglutaryl-CoA
HTN: Hypertension
ICU: Intensive care unit
IHD: Ischemic heart disease
IL-6: Interleukin-6
IRB: Institutional research board
NF-κB: Nuclear factor kappa B
NLRP3: Nod-like receptor pyrin domain containing 3
PAI-1: Plasminogen activator inhibitor-1
PCR: Polymerase chain reaction
PE: Pulmonary embolism
PFTs: Pulmonary function tests
RR: Respiratory rate
SARS: Severe acute respiratory syndrome
SARS-COV-2: Severe acute respiratory syndrome—coronavirus 2
SOFA score: Sequential organ failure assessment
