# Supplementary material for: Atorvastatin for reduction of 28-day mortality in severe and critical COVID-19 patients: a randomized controlled trial
Source: Respir Res. 2024 Feb 22;25:97. doi: 10.1186/s12931-024-02732-2 (PMC10885389; doi:10.1186/s12931-024-02732-2)
Supplement: Supplementary file 1 — Additional file 1: Table S1. Presentation on admission. Data presented as mean (SD), median (P25th–P75th), or number (%).Table S2. CRP, WHO, SOFA scores in survived in-hospital patients. Data presented as mean (SD), median (P25th–P75th), or number (%). Table S3. Drugs and Interventions Offered to patients in both groups. Table S4. 28-mortality by severity. Table S5. Six-month mortality by severity. [file 12931_2024_2732_MOESM1_ESM.docx]

| Table S1. Presentation on admission. Data presented as mean (SD), median (P_25th_–P_75th_), or number (%) | | |
| --- | --- | --- |
|  | **Group A**  **(n = 110)** | **Group B**  **(n = 110)** |
| Fever | 103 (93.6%) | 99 (90.8) |
| Cough | 99 (90%) | 96 (87.3%) |
| Sore throat | 17 (15.7%) | 12 (10.9%) |
| Dyspnea | 73% (67%) | 68 (62.4%) |
| Wheezes | 28 (25.5%) | 29 (26.6%) |
| Gastrointestinal symptoms | 25 (22.9%) | 30 (28.3%) |
| Loss of smell | 37 (33.6%) | 36 (32.7%) |
| Loss of taste | 37 (33.6%) | 33 (30%) |
| Altered Sensorium | 12 (11.9%) | 16 (14.5%) |
| Headache | 33 (30%) | 38 (34.5%) |
| Fatigue | 52 (47.3%) | 50 (45.5%) |
| Myalgia | 14 (12.7%) | 15 (13.6%) |
| Arthralgia | 3 (2.7%) | 6 (5.5%) |
| Abbreviations: Group A, Atorvastatin group; Group B, Placebo group | | |

| Table S2. CRP, WHO, SOFA scores in survived in-hospital patients. Data presented as mean (SD), median (P_25th_–P_75th_), or number (%) | | | | | |
| --- | --- | --- | --- | --- | --- |
|  | **Group A**  **(n = 110)** | **Valid**  **cases** | **Group B**  **(n = 110)** | **Valid**  **cases** | **P-value** |
| CRP 3 | 48 (16 – 119) | 59 | 48 (20.75 – 101) | 66 | .624 |
| CRP 7 | 31 (11.5 – 97.5) | 65 | 29 (12 – 96) | 72 | .525 |
| CRP 14 | 24 (6 – 92) | 23 | 12 (6 – 46) | 24 | .376 |
| CRP 28 | 96 (27 – 98) | 5 | 6 (.75 – 57) | 6 | .177 |
| SOFA 3 | 2 (1 – 3) | 108 | 2 (1 – 3) | 102 | .59 |
| SOFA 7 | 2 (1 – 5) | 86 | 2 (1 – 4) | 83 | .815 |
| SOFA 14 | 2 (1 – 6.25) | 30 | 2 (1 – 7.25) | 32 | .655 |
| SOFA 28 | 1 (.5 – 2.5) | 5 | 2 (.5 – 6) | 9 | .699 |
| WHO 3 | 5 (5 – 6) | 106 | 5 (5 – 6) | 108 | .598 |
| WHO 7 | 5 (5 – 6) | 85 | 5 (5 – 6) | 87 | .334 |
| WHO 14 | 5 (5 – 7.5) | 28 | 5 (5 – 8) | 31 | .88 |
| WHO 28 | 5 (5 – 5) | 4 | 5 (5- 5.75) | 8 | .808 |
|  |  |  |  |  |  |
| Group A, Atorvastatin group; Group B, Placebo group; CRP, C-Reactive protein; WHO, World Health Organization score; SOFA, Sequential Organ Failure Assessment score  * *P* ≤ 0.05 indicated statistical significance, # in survived patients till discharge. | | | | | |

| S3. Drugs and Interventions Offered to patients in both groups | | | |
| --- | --- | --- | --- |
|  | **Atorvastatin** | **Control** | **P value** |
| Steroids | | | 0.456 |
| No steroids | 2 | 0 |  |
| Dexamethasone | 91 | 91 |  |
| Methylprednisolone | 15 | 18 |  |
| Methylprednisolone & Dexamethasone | 2 | 1 |  |
| Anticoagulants | | | 0.326 |
| No anticoagulants | 2 | 3 |  |
| Anticoagulant not reported | 0 | 2 |  |
| anticoagulants | 108 | 105 |  |
| Immune modulator | | | 0.197 |
| Tocilizumab | 3 | 6 |  |
| Baricitinib | 1 | 0 |  |
| Oxygen therapy | | |  |
| Nasal cannula | 2 | 1 | 0.464 |
| HFNC | 55 | 42 |  |
| BIPAP/CPAP | 51 | 56 |  |
| Invasive ventilation | 38 | 43 |  |
| Abbreviations: HFNC, High Frequency Nasal Cannula; BIPAP, Bilevel Positive Airway Pressure, CPAP, Continuous Positive Airway Pressure | | | |

| **Table S4. 28-mortality by severity** | | | | |
| --- | --- | --- | --- | --- |
|  | **Group A** | **Group B** | **RR (95% CI)** | **P value** |
| **Severe cases** | 34/84 (40.5 %) | 39/86 (45.3%) | 0.893 (0.63, 1.263) | 0.521 |
| **Critical cases** | 18/20 (90%) | 15/17 (88.2%) | 1.02 (0.813, 1.28) | 0.863 |
| Group A, **Atorvastatin group**; Group B, **Placebo group** | | | | |

| **Table S5. Six-month mortality by severity** | | | | |
| --- | --- | --- | --- | --- |
|  | **Group A** | **Group B** | **RR (95% CI)** | **P value** |
| **Severe Cases** | 34/82 (41.5 %) | 44/80 (55%) | 0.754 (0.545, 1.043) | 0.085 |
| **Critical cases** | 19/20 (95%) | 15/17 (88.2%) | 1.077 (0.881, 1.316) | 0.452 |
| Group A, **Atorvastatin group**; Group B, **Placebo group** | | | | |
